# Microbial Profile of Soil-Free *versus* In-Soil Grown Lettuce and Intervention Methodologies to Combat Pathogen Surrogates and Spoilage Microorganisms on Lettuce

**DOI:** 10.3390/foods2040488

**Published:** 2013-11-11

**Authors:** Sujata A. Sirsat, Jack A. Neal

**Affiliations:** Conrad N. Hilton College of Hotel and Restaurant Management, University of Houston, Houston, TX 77204-3028, USA; E-Mail: jneal@central.uh.edu

**Keywords:** organic produce, aquaponics, microbial quality, interventions, soilless produce, lettuce, *Salmonella*, *E. coli*

## Abstract

Aquaponics is an effective method to practice sustainable agriculture and is gaining popularity in the US; however, the microbial safety of aquaponically grown produce needs to be ascertained. Aquaponics is a unique marriage of fish production and soil-free produce (e.g., leafy greens) production. Fish are raised in fresh water tanks that are connected to water filled beds where fruits and vegetables are grown. The fish bi-products create nutrient-rich water that provides the key elements for the growth of plants and vegetables. The objective of this study was to perform a comparative analysis of the microbial safety and quality of aquaponic lettuce and soil grown lettuce (conventional, bagged, certified organic, and field lettuce). Following this, an intervention study was performed to combat foodborne pathogen surrogates (*Salmonella* and *E. coli*), spoilage, and fecal microorganisms using 2.5% acetic acid. The results of the comparative analysis study showed that aquaponically grown lettuce had significantly lower concentration of spoilage and fecal microorganisms compared to in-soil grown lettuce. The intervention study showed that diluted vinegar (2.5% acetic acid) significantly reduced *Salmonella*, *E. coli*, coliforms, and spoilage microorganisms on fresh lettuce by 2 to 3 log CFU/g. Irrespective of growing methods (in-soil or soilless), it is crucial to incorporate good agricultural practices to reduce microbial contamination on fresh produce. The intervention employed in this study can be proposed to small farmers and consumers to improve quality and safety of leafy greens.

## 1. Introduction

### 1.1. Leafy Greens

Leafy greens have been growing in popularity in recent years because of the nutritional benefits and convenience as a ready-to-eat (RTE) product. However, leafy greens have been implicated in several foodborne disease outbreaks due to contamination with pathogens such as *Escherichia coli* O157:H7 [1], *E. coli* O145 [2], *Salmonella* spp [3], and norovirus [4]. Moreover, fresh produce such as lettuce and spinach cannot be processed with harsh interventions (e.g., heat, acid, *etc*.) due to loss of quality. Hence, it is especially crucial that effective pre- and post-harvest interventions be employed to reduce the risk of foodborne pathogen contamination. 

Conventional lettuce is grown in large farms using chemical pesticides [5]. Organic lettuce production is regulated by USDA’s National Organic Program (NOP) [6]. Small farmers (annual income of less than $500,000) may or may not be certified by the NOP and sell produce directly to local restaurants or consumers at the farmers’ markets. Currently, these farmers are exempt from much of the Food Safety Modernization Act (FSMA) [7]. However, they are expected to follow good agricultural practices (GAPs). Hence, it is crucial to provide these farmers with scientifically validated intervention methodologies to ensure produce safety. 

Traditional in-soil lettuce is grown outdoors where animals, insects, birds, and humans can transmit human pathogens before or during the harvesting process [8]. In addition, foodborne pathogens such as *Salmonella*, *E. coli* O157:H7 and *Listeria monocytogenes* can persist in agricultural soil for multiple days, if not longer [9,10]. *E. coli* O157:H7 populations were shown to survive in manure-amended soil for 77, 226, and 231 days for soil held at 5, 15, and 21 °C, respectively [11]. Other studies reported that *Salmonella* artificially inoculated in soil can survive for at least 45 days. In addition, *Salmonella* can survive and grow on tomatoes in contact with soil when stored at 20 °C for 4 days [12]. 

### 1.2. Aquaponics

**Figure 1:**
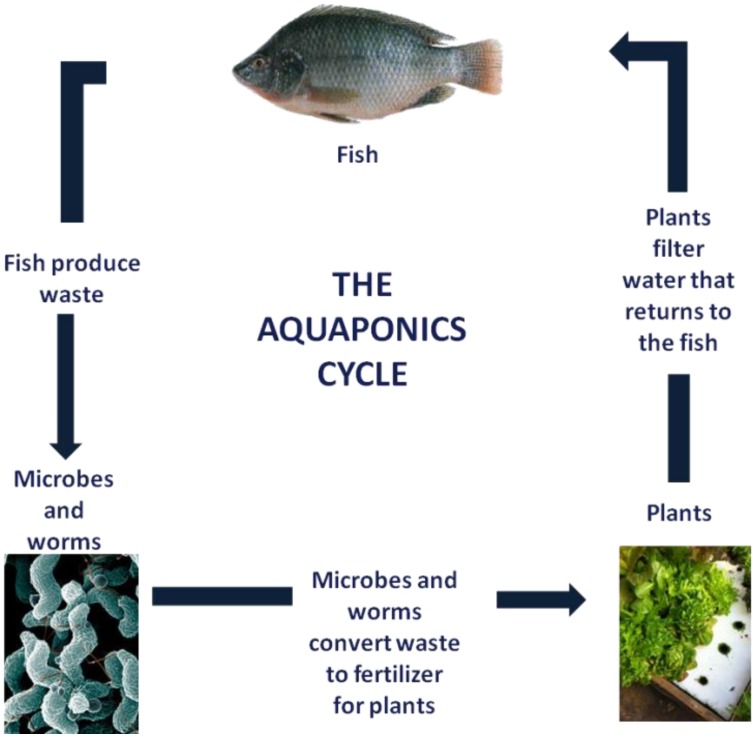
Diagrammatic representation of the aquaponic cycle.

Aquaponics is a soil-free method to practice sustainable agriculture that has gained popularity in the US. This technology involves a vegetable production system that integrates soil-free cultivation and aquaculture [13,14]. Aquaponics is a marriage of fish production (e.g., tilapia, catfish, bluegills) and soilless vegetable (e.g., lettuce, tomatoes, fresh herbs) production (Figure 1). Fish are raised in tanks and via a filtration system, are linked to water growing beds where fruits and vegetables flourish. The fish waste and uneaten foods are broken down into ammonia (NH_3_), nitrite and nitrates by beneficial bacteria. Nitrates serve as fertilizer to the plants and flourish as a result. The plants serve as filters and hence purify the water which circulates into the fish tanks [15,16]. This process renders the aquaponics system environmental-friendly since spent water and nutrients are reused. Aquaponic produce is grown in a cultured environment with no soil. However, there is no published literature on the microbial profile (fecal and spoilage microorganisms) of aquaponically grown lettuce *versus* in-soil lettuce.

### 1.3. Interventions

It is crucial to incorporate on-farm GAPs and post-harvest processing intervention steps for fresh produce irrespective of growing methodologies to ensure produce safety. This study focused on post-harvest processing and screened a variety of novel antimicrobials that can be proposed to small farmers to improve safety and quality of fresh produce. A farmer survey demonstrated that most of the farmers do not wash fresh produce and those who do use only potable water [17]. Water alone may not be as effective as an antimicrobial intervention in reducing the prevalence of foodborne pathogens on produce [18]. Hence, the purpose of this study was to propose natural antimicrobial interventions that small farmers can employ to keep produce safe and reduce the risk of foodborne pathogens. Acetic acid (white vinegar) is a natural compound, easily accessible, and inexpensive. Previous studies have shown that acetic acid is effective against foodborne pathogens [19,20,21]. 

The objectives of this study were three-fold: (1) compare the microbial profile (coliforms, *E. coli*, yeast, and mold) of aquaponic lettuce to in-soil (organic, conventional, bagged, and field) lettuce; (2) investigate the effects of acetic acid at different concentrations and contact times on *Salmonella* and *E. coli* inoculated on fresh leafy greens; and (3) test the efficacy of the most effective concentration and contact time of the natural intervention against fecal and spoilage microorganisms present on lettuce. Overall, this study aims to obtain a better understanding of the microbial profile of various types of lettuce and propose a scientifically validated methodology to farmers and consumers to reduce the risk of foodborne illness and improve shelf-life.

## 2. Experimental Section

### 2.1. Sampling

#### 2.1.1. Obtaining Leafy Green Samples

Conventional romaine, certified organic romaine, and bagged romaine lettuce, typical of those entering the U.S. food supply, were purchased from a major retail supplier in the Houston, TX area. It should be noted that in this study, the investigators describe “field lettuce” as lettuce that is most often sold by small farmer vendors at farmers’ market. This lettuce has not been exposed to chemicals during pre- or post-harvest. The investigators obtained this lettuce from four different small farm located around the Houston, TX area and purchased from the farmers market. One aquaponic green-house facilities located at the Houston Community College (Houston, TX, USA) kindly provided aquaponically grown soil-free lettuce. The aquaponic and field lettuce samples were obtained directly from the growers. The other lettuce samples (conventional, organic, and bagged) were obtained from the grocery store. The samples were handled aseptically and stored at 4 °C and transported to the University of Houston Food Safety Laboratory. The lettuce was collected from various sources over a three-month period. As soon as the lettuce was purchased, it was transported to the laboratory and placed in the refrigerator and processed within a 12-h period. A total of 12 samples were analyzed for each lettuce type. Three biological replicates were performed for each sample.

#### 2.1.2. Processing Samples for Microbial Quality Analysis

Lettuce samples weighing 10 g were aseptically added to sterile stomacher bags containing 90 mL 0.1% peptone buffer. The samples were stomached for 120 s in a stomacher and appropriate dilutions were performed and microbial analysis was carried out. 

#### 2.1.3. Microbial Analysis

The 3M petrifilms™ (St. Paul, MN, USA) were used for aerobic plate counts (APC) and coliform enumeration. One milliliter samples were plated on aerobic pertrifilms and coliform plates to quantify all aerobic bacteria, coliforms and *E. coli*. The APC and coliform petrifilms were incubated in a 35 °C and 37 °C incubator respectively. Potato dextrose media (Becton, Dickinson and Company Diagnostics, Franklin Lakes, NJ, USA) with chloramphenicol (Amresco, Solon, OH, USA; PDMC) was prepared to quantify yeast and mold and prevent the growth of bacteria. The PDMC plates were incubated at room temperature for 72 to 96 h for yeast and mold quantification. Three biological replicates were performed for each sample.

### 2.2. Intervention

#### 2.2.1. Pathogen Surrogate Growth Conditions and Lettuce Inoculation

*Salmonella* Typhimurium ATCC 53647, *E. coli* ATCC 25922, and *E. coli* ATCC 10798 (*E. coli* K12) used in this study was obtained from American Type Culture Collection (Manassas, VA, USA) and stored at −80 °C in glycerol. The cultures were streaked on Brain Heart Infusion (BHI) agar (Becton, Dickinson and Company Diagnostics, Franklin Lakes, NJ, USA) and incubated at 37 °C overnight. Single colonies were used to inoculate BHI broth (Becton, Dickinson and Company Diagnostics, Franklin Lakes, NJ, USA) and grown at 37 °C for 18 h and used to make a cocktail prior to the experiment. The bacterial cocktail was inoculated on lettuce to obtain a final concentration of approximately 10^8^ CFU/g lettuce and allowed to air dry for 30 min.

#### 2.2.2. Intervention Study

Preliminary studies were carried out to identify the most effective concentration and contact time for acetic acid against the pathogen surrogates inoculated on lettuce. This was done by screening white vinegar, apple cider vinegar, red wine vinegar and lemon juice (all purchased from a local supermarket chain). *E. coli* and *Salmonella* cocktails were added to undiluted interventions and incubated for 0, 30, and 60 min. Following this, white vinegar (which was the most effective antimicrobial) was studied at 5%, 2.5%, and 1.25% for 30, 60, and 120 s on the lettuce samples inoculated with the bacterial cocktail. Pre-weighed lettuce samples (10 g) were added to a salad spinner with the vinegar and dipped for appropriate times. The samples were transferred to sterile stomacher bags and stomached with 0.1% peptone water for 120 s. Appropriate dilutions were performed in 0.1% peptone water (pH 7) and the samples were plated on Eosin Methylene Blue (EMB) agar (Becton, Dickinson and Company Diagnostics, Franklin Lakes, NJ, USA). Following this, the most effective contact time and acetic acid concentration was tested against fecal and spoilage microorganisms found on lettuce. Analysis was done pre- and post-intervention to detect the concentration of fecal and spoilage microorganisms on the lettuce. The analysis was conducted as described above and samples were plated on 3M petrifilms™ for APC, coliform and *E. coli* analyses and PDMC plates for yeast and mold enumeration. Three biological replicates were performed for all the intervention experiments.

### 2.3. Statistical Analysis

Microbiological data was converted to log CFU/cm^2^ and one-way analysis of variance (ANOVA) was calculated using IBM SPSS 19 software. Tukey’s HSD (honestly significant difference) Post-hoc tests were used to compare mean log CFU/g values. *P* values ≤ 0.05 were considered statistically significant. The detection limit of this study was 1 log CFU/cm^2^. All samples below detection limit were assigned a value of 0.1. The differences between before and after intervention treatments were analyzed using Microsoft Excel (Microsoft Office 2007). Type 2 *t*-tests statistics were performed to test significant differences among different groups.

## 3. Results and Discussion

**Figure 2:**
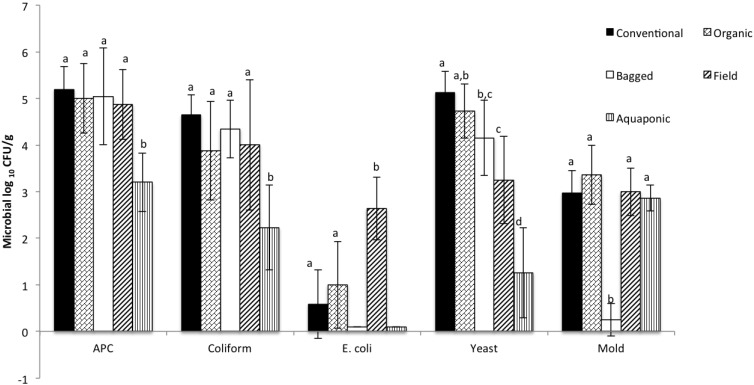
Comparative microbial analysis of conventional, organic, bagged, farmers’ market, and aquaponic lettuce. Bars represent mean aerobic plate counts (APC), coliform, *E. coli*, yeast, and mold counts (log CFU/g lettuce). Error bars represent standard deviations from the mean. Statistical differences are shown using letters.

The results showed that there was a significant difference in APC and coliform counts between aquaponic and all other lettuce types (Figure 2). No detectable *E. coli* were observed in bagged or aquaponic lettuce samples. Lettuce from farmers’ markets contained 2 to 3.5 log CFU/g *E. coli*. Both conventional and organic lettuce contained approximately 2 log CFU *E. coli* per gram of lettuce. These results are comparable to studies performed by the FDA to identify pathogenic *E. coli* in domestic produce [22]. Yeast counts were highest for conventional and organic lettuce (5.5 and 5 log CFU/g respectively). Aquaponic lettuce contained approximately 2 to 3 log CFU/g fewer yeast compared to other lettuce types (significant at *p* < 0.05). Bagged lettuce samples had significantly lower mold counts compared to all other lettuce types. Only 17% of bagged lettuce samples were positive for mold.

In a different study, a group of researchers sampled fresh produce and tested the microbial quality (*Escherichia coli*, total aerobic bacteria, total coliforms, and total Enterococcus) at the farm and packing shed. Results showed that microbial counts increased as the produce was transferred from farm to the packing shed during harvest. The results in the current study follow a similar trend as shown by Ailes (2008) [23]. Aquaponic leafy greens and field greens were sampled directly from the greenhouse and farm respectively. Hence, these samples were minimally processed. However, other leafy greens sampled (organic, conventional, and bagged) underwent the harvesting, post-processing (possibly chemical interventions), packaging, and transport stages. As seen in Figure 2, a significant difference in APC, coliform, *E. coli*, and yeast counts was observed between field and aquaponic lettuce.

There is very limited literature on the microbiological safety of aquaponically grown produce. Previous studies by Selma *et al.* [24] reported sensory and microbial differences between soil and other types of soilless agriculture. The researchers used sandy clay loom in accordance to the USDA guidelines to develop a soilless system to grow produce. These results showed that a soilless system had higher sensory quality and had a lower number of spoilage microorganisms compared to in-soil produce. The results presented by Selma *et al.* [12] are comparable to the results presented in the current study. Independent studies have demonstrated comparisons between organic and conventional lettuce varieties in Brazil [25]. The microbial profiles and counts (*E. coli*, yeast and mold) for conventional and organic lettuce were confirmed by the current study. Another study tested the microbial quality of bagged lettuce and found up to 7 log CFU/g total APC microorganisms [26]. Previous literature also shows that there may be a higher bacterial count on the lettuce sampled from the bottom compared to the top of the bag [26,27]. We sampled lettuce from the top and bottom of the bag and found no significant differences in microbial counts (data not shown). 

**Figure 3:**
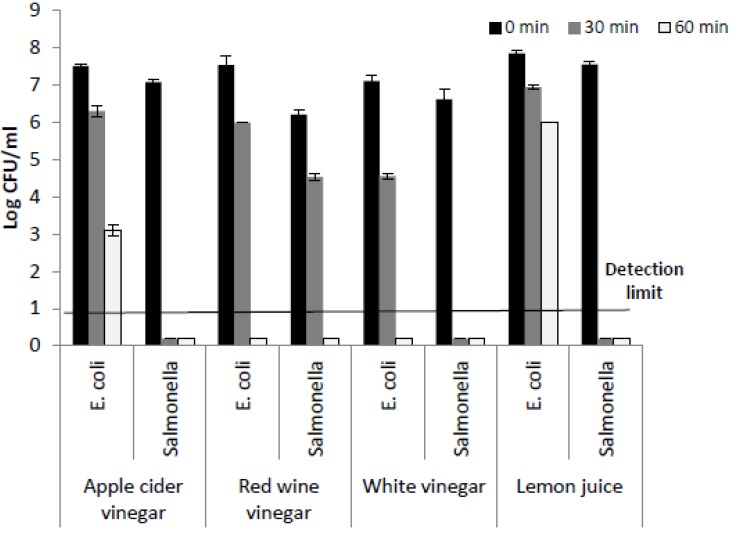
Efficacy of various undiluted vinegar types (white (pH 2.4), apple cider (pH 3.10), red wine (pH 2.73) and undiluted lemon juice (pH 2.57) on *E. coli* and *Salmonella in vitro*.

In addition to the comparative microbial quality analysis, a natural intervention protocol was employed on leafy greens to improve overall quality and reduce spoilage microorganisms. For this study, a number of natural agents such as vinegars (white, red wine, and apple cider) and lemon juice were screened for their efficacy against *Salmonella* and *E. coli in vitro*. White vinegar was the most effective antimicrobial against the foodborne pathogen surrogates (Figure 3). Following this, various concentrations of white vinegar were used against a cocktail of pathogen surrogates inoculated on lettuce. Vinegar at 1.25%, 2.5%, and 5% acetic acid concentration significantly reduced *Salmonella* and *E. coli* viability as compared to control (water) at *p* < 0.05 (Table 1). White vinegar at 2.5% and 5% had a similar effect on bacterial viability at 60 s. Hence, the lower concentration of acetic acid was chosen as most effective and used for further experiments.

**Table 1:** Log reductions demonstrating acetic acid intervention at various concentrations and contact times against *E. coli* and *Salmonella* (pH 1.25% acetic acid = 2.8; 2.5% acetic acid = 2.6; 5% acetic acid = 2.4).

1.25 % Acetic Acid		2.5% Acetic Acid		5% Acetic Acid	
*E. coli*	*Salmonella*	*E. coli*	*Salmonella*	*E. coli*	*Salmonella*
0.73 ± 0.23	1.55 ± 0.23	1.43 ± 0.15	1.36 ± 0.33	0.13 ± 0.15	0.36 ± 0.05

White vinegar has been demonstrated to have strong antimicrobial properties due to its high acetic acid content [19,20]. Previous studies have demonstrated that vinegar is especially effective against *Salmonella choleraesuis* and *Pseudomonas aeruginosa* [21]. Other studies compared the bactericidal abilities of vinegar, olive oil, and other compounds against various pathogenic strains of *E. coli*, *Salmonella*, *Listeria*, *Yersinia*, and *Staphylococcus* [28]. The results demonstrated that vinegar reduced the counts of inoculated *L. monocytogenes*, *Salmonella* Enteritidis, *S. sonnei*, and *Yersinia* sp. to levels below the detection limit and killed most of the *E. coli* and *S. aureus* cells. 

Results from the current study showed the efficacy of vinegar against not only foodborne pathogen surrogates, but also spoilage microorganisms. The most effective concentration and contact time (2.5% at 60 s) was applied against spoilage and coliform microorganisms on lettuce. A significant difference (*p* < 0.05) was observed for APC, coliform, *E. coli*, yeast and mold count after intervention (Table 2). Hence, the application of this natural intervention agent may combat pathogenic and spoilage microorganisms leading to reduced risk of foodborne illness and improved shelf-life.

**Table 2:** Logarithmic reductions following an acetic acid intervention at various concentrations and contact times against spoilage and fecal microorganisms present on lettuce (APC, coliforms, *E.coli*, yeast and mold).

Aerobic Plate Counts	Coliforms	*E. coli*	Yeast	Mold
2.18 ± 0.18	2.5 ± 0.51	1.46 ± 1.51	3.7 ± 1.4	2.9 ± 0.4

This study investigated the microbial of profile of aquaponically grown lettuce in comparison to different types of traditionally grown (in-soil) lettuce. To our knowledge, this type of investigation has not been documented in the literature. Aquaponic technology is commonly practiced in countries such as Thailand and Australia and is currently gaining popularity in the US. Irrespective of produce growing methods, good agricultural practices (GAPs) and good pre- and post-harvest methods need to be employed to ensure microbial food safety. For this, the current study investigated a variety of natural sanitizer agents that can be employed by small farmers to rinse lettuce and reduce the prevalence of microbial foodborne contamination.

## 4. Conclusions

Aquaponically grown lettuce had significantly lower concentration of microorganisms compared to other in-soil grown lettuce. The results from this study suggest that aquaponic produce may have a lower risk for foodborne illness and an improved shelf-life, due to its low bacterial counts compared to soil grown produce. These results are not unexpected since the aquaponic system used for this study was held in a controlled and soil-free green-house environment. In addition, the intervention results demonstrated that a simple vinegar wash is effective against pathogenic and spoilage microorganisms. Vinegar is a natural and readily available agent. In the future, the antimicrobial intervention can be proposed to small farmers and possibly consumers to improve the microbial quality and safety of fresh produce. 

## References

[1] Investigation Update: Multistate Outbreak of *E. coli* O157:H7 Infections Linked to Romaine Lettuce. http://www.cdc.gov/ecoli/2011/ecoliO157/romainelettuce/032312/index.html.

[2] Investigation Update: Multistate Outbreak of Human *E. coli* O145 Infections Linked to Shredded Romaine Lettuce from a Single Processing Facility. http://www.cdc.gov/ecoli/2010/ecoli_o145/index.html.

[3] Lienemann T., Niskanen T., Guedes S., Siitonen A., Kuusi M., Rimhanen-Finne R. (2011). Iceberg lettuce as suggested source of a nationwide outbreak caused by two *Salmonella* serotypes, Newport and Reading, in Finland in 2008. J. Food Prot..

[4] Outbreaks of Gastroenteritis Linked to Lettuce. http://www.eurosurveillance.org/images/dynamic/EE/V15N06/art19484.pdf.

[5] Oliveira M., Usall J., Viñas I., Anguera M., Gatius F., Abadias M. (2010). Microbiological quality of fresh lettuce from organic and conventional production. Food Microbiol..

[6] National Organic Program. http://www.ams.usda.gov/AMSv1.0/ams.fetchTemplateData.do?template=TemplateA&navID=NationalOrganicProgram&leftNav=NationalOrganicProgram&page=NOPNationalOrganicProgramHome&acct=AMSPW.

[7] Food Safety Modernization Act. http://www.fda.gov/Food/FoodSafety/FSMA/default.htm.

[8] Kader A.A. (2002). Postharvest Technology of Horticultural Crops.

[9] Stenstrom T.A. (1989). Bacterial hydrophobicity, an overall parameter for the measurement of adhesion potential to soil particles. Appl. Environ. Microbiol..

[10] Wei J., Jin Y., Sims T., Kniel K.E. (2011). Murine norovirus-1 internalization into *Lactuca sativa* during irrigation. Appl. Environ. Microbiol..

[11] Jiang X., Morgan J., Doyle M.P. (2002). Fate of *Escherichia coli* O157:H7 in manure-amended soil. Appl. Environ. Microbiol..

[12] Guo X., Chen J., Brackett R.E., Beuchat L.R. (2002). Survival of *Salmonella* on tomatoes stored at high relative humidity, in soil, and on tomatoes in contact with soil. J. Food Prot..

[13] A Preliminary Study of Microbial Water Quality Related to Food Safety in Recirculating Aquaponic Fish and Vegetable Production Systems. http://www.ctahr.hawaii.edu/oc/freepubs/pdf/FST-51.pdf.

[14] On-Farm Food Safety: Aquaponics. http://www.ctahr.hawaii.edu/oc/freepubs/pdf/FST-38.pdf.

[15] Recirculating Aquaculture Tank Production Systems: Aquaponics—Integrating Fish and Plant Culture. http://www2.ca.uky.edu/wkrec/454fs.PDF.

[16] Goodman E.R. (2011). Aquaponics: Community and Economic Development. Masters Thesis.

[17] Zerio-Egli C. (2012). Economical Post-Harvest Practices for Leafy Greens Grown on Texas Small Farms. Master’s Thesis.

[18] Guidance for Industry. Guide to Minimize Microbial Food Safety Hazards for Fresh Fruits and Vegetables. http://www.fda.gov/downloads/Food/GuidanceComplianceRegulatoryInformation/GuidanceDocuments/ProduceandPlanProducts/UCM169112.pdf.

[19] Entani E., Asai M., Tsujihata S., Tsukamoto Y., Ohta M. (1998). Antibacterial action of vinegar against food-borne pathogenic bacteria including *Escherichia coli* O157:H7. J. Food Prot..

[20] Wu F., Doyle M., Beuchat L., Wells J., Mintz E., Swaminathan B. (2000). Fate of *Shigella sonnei* on parsley and methods of disinfection. J. Food Prot..

[21] Rutala W.A., Barbee S.L., Aguiar N.C., Sobsey M.D., Weber D.J. (2000). Antimicrobial activity of home disinfectants and natural products against potential human pathogens. Infect. Control Hosp. Epidemiol..

[22] FDA Survey of Domestic Fresh Produce-Domestic Produce Assignment. http://www.fda.gov/Food/GuidanceRegulation/GuidanceDocumentsRegulatoryInformation/ProducePlantProducts/ucm118297.htm.

[23] Ailes E.C., Leon J.S., Jaykus L.A., Johnston L.M., Clayton H.A., Blanding S., Kleinbaum D.G., Backer L.C., Moe C.L. (2008). Microbial concentrations on fresh produce are affected by postharvest processing, importation, and season. J. Food Prot..

[24] Selma M.V., Luna M.C., Martínez-Sánchez A., Tudela J.A., Beltrán D., Baixauli C., Gil M.I. (2012). Sensory quality, bioactive constituents and microbiological quality of green and red fresh-cut lettuces (*Lactuca sativa* L.) are influenced by soil and soilless agricultural production systems. Postharvest Biol. Technol..

[25] Maffei D.F., de Arruda Silveira N.F., Catanozi M.P.L.M. (2012). Microbiological quality of organic and conventional vegetables sold in Brazil. Food Control.

[26] Valentin-Bon I., Jacobson A., Monday S.R., Feng P.C.H. (2008). Microbiological quality of bagged cut spinach and lettuce mixes. Appl. Environ. Microbiol..

[27] Kase J.A., Borenstein S., Blodgett R.J., Feng P.C.H. (2012). Microbial quality of bagged baby spinach and romaine lettuce: Effects of top *versus* bottom sampling. J. Food Prot..

[28] Medina E., Romero C., Brenes M., de Castro A. (2007). Antimicrobial activity of olive oil, vinegar, and various beverages against foodborne pathogens. J. Food Prot..

